# Supplementary figures and images for: Research on vehicle lateral stability control under low-adhesion road conditions using proximal policy optimization algorithm
Source: PLoS One. 2025 Nov 26;20(11):e0335686. doi: 10.1371/journal.pone.0335686 (PMC12654942; doi:10.1371/journal.pone.0335686)

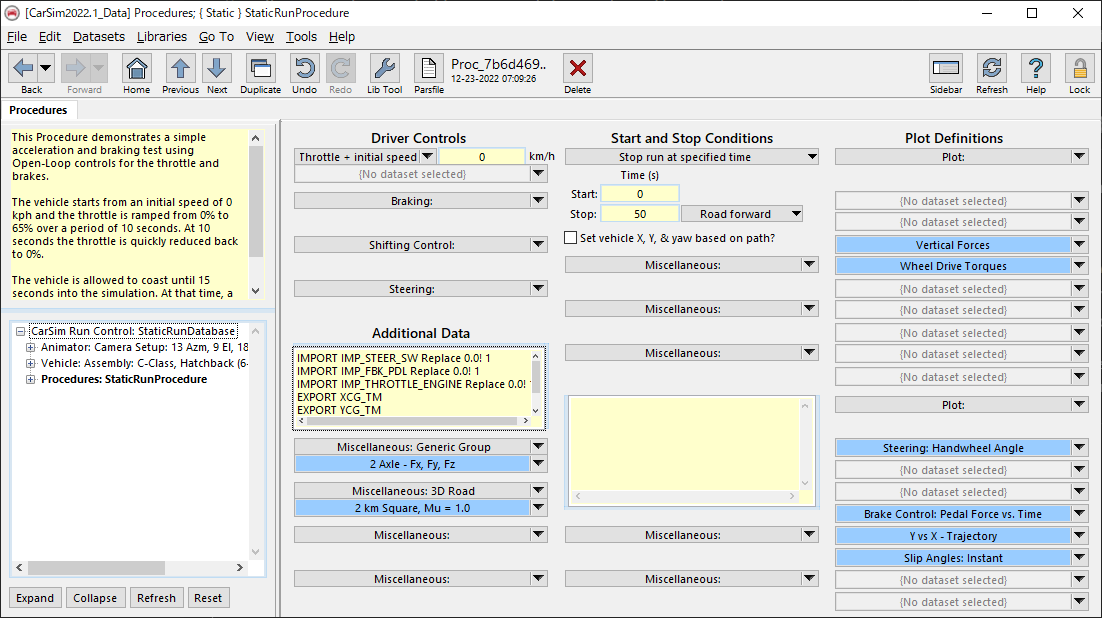

Supplement: S2 File — (ZIP) [file pone.0335686.s002.zip › Main_PPO/pycarsimlib-master/media/carsim_procedures_window.png]

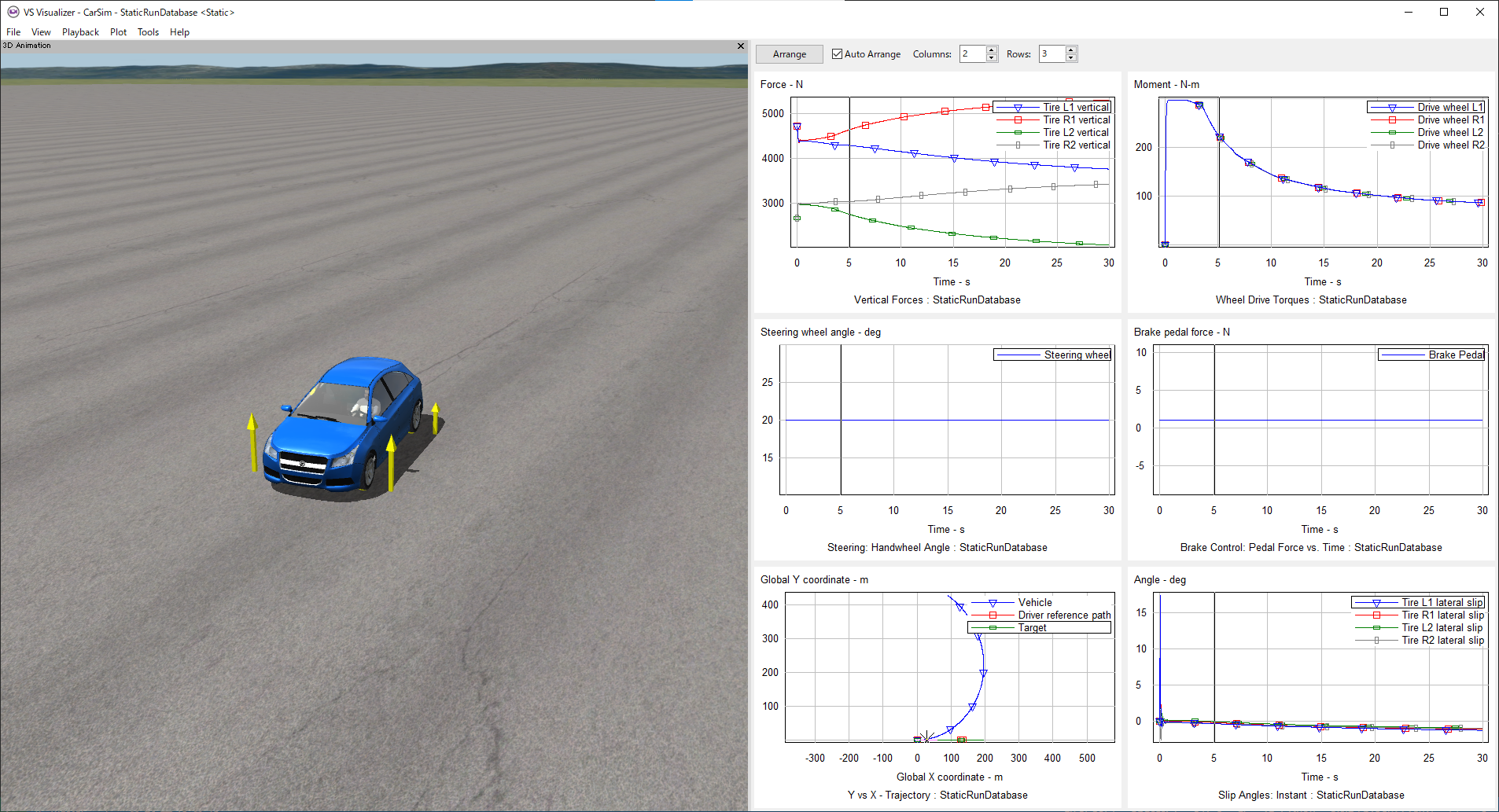

Supplement: S2 File — (ZIP) [file pone.0335686.s002.zip › Main_PPO/pycarsimlib-master/media/carsim_visualizer_window.png]

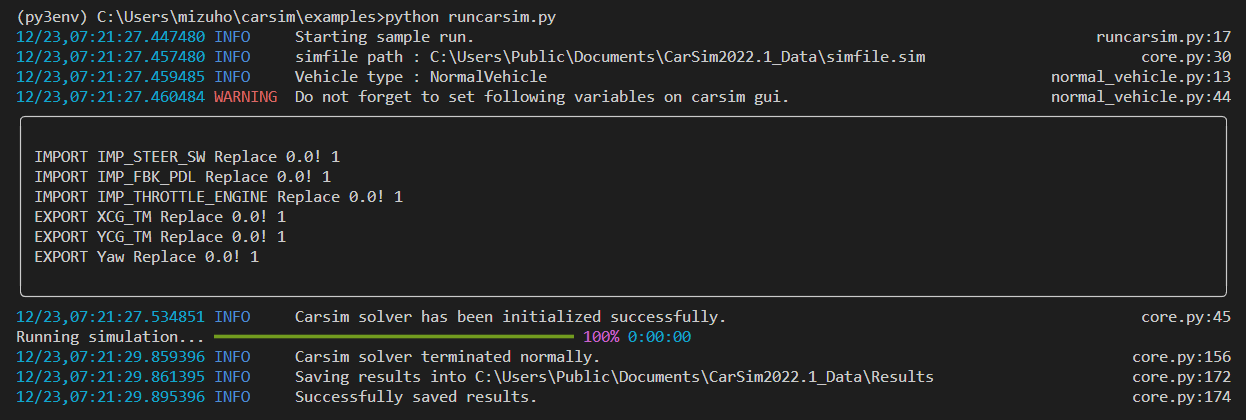

Supplement: S2 File — (ZIP) [file pone.0335686.s002.zip › Main_PPO/pycarsimlib-master/media/runcarsim_shell_output.png]
