# Supplementary material for: Research on vehicle lateral stability control under low-adhesion road conditions using proximal policy optimization algorithm
Source: PLoS One. 2025 Nov 26;20(11):e0335686. doi: 10.1371/journal.pone.0335686 (PMC12654942; doi:10.1371/journal.pone.0335686)
Supplement: Attachment — Submitted filename: Response to Reviewers.pdf [file pone.0335686.s004.pdf]

Editor and Reviewer Comments:

We would like to express our gratitude to the academic editor and reviewers for their valuable comments. We have revised the manuscript accordingly, and our point-by-point responses to each comment from the academic editor and reviewers are provided below.

Additional Editor Comments:

Please revise the manuscript thoroughly according to the reviewers' comments. In addition, the literature review on vehicle lateral stability control should incorporate more recent studies, such as "A Robust Dynamic Game-Based Control Framework for Integrated Torque Vectoring and Active Front-Wheel Steering System, IEEE Transactions on Intelligent Transportation Systems, vol. 24, no. 7, pp. 7328-7341, July 2023", and "A Direct Yaw Moment Control Framework Through Robust T-S Fuzzy Approach Considering Vehicle Stability Margin, IEEE/ASME Transactions on Mechatronics, vol. 29, no. 1, pp. 166-178, Feb. 2024".

Answer question: It has been revised, line on 69-149 (in Manuscript); line on 73-161 (in Revised Manuscript with Track Changes). The newly added references include [3], [11-17], etc.

Reviewer #1: This paper proposes to use PPO deep reinforcement learning to achieve longitudinal and horizontal cooperative stability control of low-attached pavement, and verifies it by Amesim-CarSim-Python co-simulation and HIL experiments. The overall idea is clear, the experiment is sufficient, and the results are credible. However, there are still some problems, it is recommended to modify and adopt, see the following for the specific problems:

1.The limitations of deep reinforcement learning (DRL) (safety, interpretability, training costs) are not mentioned. The authors should explain these limitations in a few sentences.

Answer question: It has been revised, line no 144-149 (in Manuscript); line no 156-161 (in Revised Manuscript with Track Changes).

2.There is no uniform definition of "low-adhesion road surface." Line 122 of the manuscript states that $\mu < 0.3$, but line 596 states that $\mu = 0.2$. It is recommended that the definition of "low friction" be standardized.

Answer question: It has been revised, line no 156 (in Manuscript); line no 168 (in Revised Manuscript with Track Changes).

3.The symbols $\varepsilon 1$ and $\varepsilon 2$ in formula (13) have no values or references. The author needs to provide reference values or explanations.

Answer question: It has been revised, line no 369-376 (in Manuscript); line no 397-404 (in Revised Manuscript with Track Changes).

4.The parameters in Table 4 do not have physical dimensions. The author should explain this to prevent misunderstanding by readers.

Answer question: It has been revised, line no 552 (in Manuscript); line no 586 (in Revised Manuscript with Track Changes). The reward function values in the PPO (Proximal Policy Optimization) algorithm are designed as dimensionless parameter values. This characteristic stems

from the demands for algorithmic generality in reinforcement learning, the essence of optimization objectives, the requirements for numerical computational stability, and the algorithm's control mechanisms over policy updates.

5.Table 4 ω_1=-2 may make the reward value negative, and its physical meaning needs to be explained, does this punish excessive braking?

Answer question: It has been revised, line no 517-526 (in Manuscript); line no 550-559 (in Revised Manuscript with Track Changes).

6.The PPO does not specify the network structure, activation function, observation normalization method, random seed, or training-testing data division ratio, making it difficult to reproduce. The authors should supplement the table with an explanation of the number of network layers, number of nodes, and training-testing data division ratio.

Answer question:

(1)The network structure, activation function: It has been revised, line no 484-503 (in Manuscript); line no 516-535 (in Revised Manuscript with Track Changes).

(2)Random seed, Discount factor: It has been revised, line no 585 (in Manuscript); line no 619 (in Revised Manuscript with Track Changes).

(3)Observation normalization method: This study employs the PPO2 algorithm, No normalization was involved in this study.

PPO2 reduces explicit normalization reliance via clipped objectives, trust-region updates, and GAE. For simple tasks or well-designed rewards, normalization can be skipped. Its core stability mechanisms include: clipping policy updates to limit changes and mitigate gradient issues; enforcing small policy differences to reduce variance and sensitivity to extreme values; decoupling value/policy functions, with gradient clipping controlling fluctuations; and using GAE to attenuate long-term errors.

(4)Training-testing data division ratio: No training-testing data division ratio was involved in this study.

The PPO2 algorithm does not require the setting of a training-test data split ratio. This is because its data is dynamically generated, its training objective is to maximize cumulative rewards, it achieves online evaluation through environment interaction, its stability mechanisms reduce the risk of overfitting, and its evaluation metrics directly rely on environmental feedback. These characteristics enable PPO2 to train without relying on an independent test set, instead ensuring correct policy optimization direction through continuous interaction and stability design.

7.Reference 26 contains an error in the DOI number (2000-01-1060 corresponds to 2001-01-1060).

Answer question: It has been revised, line no 1002 (in Manuscript); line no 1059 (in Revised Manuscript with Track Changes).

8.The overall clarity of the picture is not high enough, and the resolution of each schematic needs to be improved.

Answer question: It has been revised.

Reviewer #2: This manuscript presents a study on an integrated longitudinal and lateral vehicle stability control system using the Proximal Policy Optimization (PPO) deep reinforcement learning algorithm. The simulation experiments verified the effectiveness of the proposed algorithm. Some issues need to be resolved before the manuscript can be accepted.

1. A more detailed description of the hyperparameter tuning methodology is recommended.

Answer question: It has been revised, line no 560-581 (in Manuscript); line no 593-615 (in Revised Manuscript with Track Changes).

2. The manuscript currently lacks sufficient comparisons with other learning algorithms to highlight the superiority of the proposed method.

Answer question:Thank you for your valuable comment regarding the comparison with other learning algorithms. First, the core research focus of this study is the application of the PPO algorithm in vehicle lateral stability control under low-adhesion road conditions. Our research work has been systematically designed around the vehicle dynamics modeling, reward function design, control logic optimization, and performance verification of the PPO algorithm. Due to the fundamental differences in the structural characteristics, hyperparameter tuning rules, and control objective adaptation mechanisms among different DRL algorithms (e.g., DDPG, TD3, TRPO), conducting rigorous comparative experiments would require in-depth research on each of these algorithms—including optimizing their hyperparameters for the specific scenario of low-adhesion road emergency braking, verifying the rationality of their action/state space design, and ensuring the consistency of evaluation metrics. Currently, our research team has not yet completed such systematic research on other DRL algorithms, and hasty comparisons without sufficient parameter optimization and scenario adaptation may lead to biased conclusions that fail to reflect the true performance of each algorithm, which is inconsistent with the rigorous requirements of academic research. If it is possible, we will implement this work in the later stage.

3. It is recommended that the authors conduct a thorough discussion on the limitations of the current research and provide an evaluation of the feasibility of deploying it on real automotive electronic control units.

Answer question:

(1) The limitations of the current research: It has been revised, line no 846-862 (in Manuscript); line no 902-918 (in Revised Manuscript with Track Changes).

(2) An evaluation of the feasibility of deploying it on real automotive electronic control units: It has been revised, line no 800-817 (in Manuscript); line no 850-867 (in Revised Manuscript with Track Changes).

4. The problem of vehicle lateral control is a well-established example of motion control for systems under nonholonomic constraints, a field with extensive research. It is suggested that the introduction incorporate more key studies on the lateral control of nonholonomic systems. Doing so would not only enrich the literature review but also help to define the unique contributions of this study and underscore the significance of tackling this classic problem with advanced reinforcement learning techniques more sharply.

a) "Paired Interactions of Magnetic Millirobots in Confined Spaces Through Data-Driven Disturbance Rejection Control Under Global Input," IEEE/ASME Transactions on Mechatronics, doi: 10.1109/TMECH.2024.3521085.

b) "A Robot Motion Learning Method Using Broad Learning System Verified by Small-Scale

Fish-Like Robot," in IEEE Transactions on Cybernetics, vol. 53, no. 9, pp. 6053-6065, Sept. 2023, doi: 10.1109/TCYB.2023.3269773.

Answer question: It has been revised, references: [13][16].

5. The manuscript is generally well-written, but a few minor grammatical checks and rephrasing could improve readability. For example, some sentences are quite long and could be broken down.

Answer question: It has been revised.

6. The manuscript's images are blurry and high-resolution versions should be provided.

Answer question: It has been revised.
